# Feeling before knowing why: The role of the orbitofrontal cortex in intuitive judgments—an MEG study

**DOI:** 10.3758/s13415-014-0286-7

**Published:** 2014-05-01

**Authors:** Ninja K. Horr, Christoph Braun, Kirsten G. Volz

**Affiliations:** 1 Werner Reichardt Center for Integrative Neuroscience, University of Tübingen, Otfried-Müller-Straße 25, Tübingen, 72076 Germany; 2 MEG-Center, University of Tübingen, Tübingen, Germany; 3 CIMeC, Center for Mind and Brain Sciences, University of Trento, Trento, Italy; 4 Research Center for Computational Neuroscience and Cognitive Robotics, University of Birmingham, Birmingham, UK

**Keywords:** Decision making, Intuition, Coherence judgments, Orbitofrontal cortex, Magnetoencephalography

## Abstract

In theory, intuitive decisions are made immediately, without conscious, reasoned thought. They are experienced as decisions based on hunches that cannot be explicitly described but, nevertheless, guide subsequent action. Investigating the underlying neural mechanisms, previous research has found the orbitofrontal cortex (OFC) to be crucial to intuitive processes, but its specific role has remained unclear. On the basis of a two-stage conceptualization of intuition suggested by Bowers, Regehr, Balthazard, and Parker *Cognitive Psychology*, 22, 72-110 (1990), we attempt to clarify the OFC’s role in intuitive processing. We propose that it functions as an early integrator of incomplete stimulus input guiding subsequent processing by means of a coarse representation of the gist of the information. On the subjective level, this representation would be perceived as a (gut) feeling biasing the decision. Our aim in the present study was to test this neural model and rule out alternative explanations of OFC activation in intuitive judgments. We used magnetoencephalography (MEG) to record participants' electromagnetic brain responses during a visual coherence judgment task. As in earlier studies, the OFC was found to be activated when participants perceived coherence. Using MEG, it could be shown that this increase in activation began earlier in the OFC than in temporal object recognition areas. Moreover, the present study demonstrated that OFC activation was independent of physical stimulus characteristics, task requirements, and participants’ explicit recognition of the stimuli presented. These results speak to the OFC’s fundamental role in the early steps of intuitive judgments and suggest the proposed neural model as a promising starting point for future investigations.

## Introduction

In everyday life, people commonly have to decide quickly between multiple alternatives, the potential consequences of which are often unpredictable. Thus everyday-life decisions are often made on the basis of incomplete stimulus information, and usually within time limitations. Such “decisions under uncertainty” stand in contrast to so-called “decisions under risk,” in which all possible alternatives and outcomes, as well as their probabilities, are known (Knight, 1921). In order to deal with decisions under uncertainty in real-life situations, an individual needs to have rapid judgmental abilities that do not depend on a conscious thought process moving through all the steps of reasoning. These kinds of rapid judgments have been termed *intuitive* (e.g., Evans, 2008; Kruglanski & Gigerenzer, 2011).

An empirically fruitful working definition for intuitive processes has been put forward by Bowers, Regehr, Balthazard, and Parker (1990), who conceive of intuition as a preliminary perception of coherence that guides further thought and action toward a hypothesis on the nature of the coherence in question. This framework suggests that intuition is a continuous process of accumulating clues of coherence from incomplete stimulus input. This accumulation, in turn, activates related mnemonic networks. In a first stage, which Bowers and colleagues termed the *guiding stage*, the accumulation of clues of coherence eventually leads to a feeling of coherence. The feeling of coherence is already strong enough to trigger subsequent decision and action, despite the fact that, in the guiding stage, it is not—or not yet—possible to explicitly report what makes the stimulus coherent. Decisions based on such initial feelings of coherence are termed *intuitive*. In a second stage, called the *integrative* stage, cues are further accumulated, and the initial hunch of coherence evolves into an explicit representation. This explicit representation “occurs when sufficient activation has accumulated to cross a threshold of awareness” (Bowers et al., 1990, p. 74), thereby making it possible for the individual to consciously reason out the decision made or action taken in the guiding stage.

Bowers and colleagues (1990) created several paradigms to investigate intuition in the context of coherence judgments. In the present study, we used a paradigm based on their Waterloo Gestalt closure task (WGCT) in order to operationalize intuitive processes in the domain of visual detection. In the WGCT, participants have to judge the coherence of incomplete line drawings that are either fragmented or scrambled. For fragmented line drawings, a certain amount of pixel information (randomly spread across the entire stimulus) is taken away, making the recognition of the originally displayed object more difficult but not impossible. In scrambled line drawings, the pixel information of the resulting fragments are, on top of that, randomly mixed up, which makes the original displayed object unrecognizable. Studies applying WGCT-like paradigms (Bolte & Goschke, 2008; Bowers et al., 1990; Luu et al., 2010; Topolinski & Strack, 2009; Volz & von Cramon, 2006) have revealed that participants discriminate between fragmented and scrambled line drawings over chance; that is, they are significantly more likely to rate fragmented than scrambled line drawings as coherent. This is true even for those coherence judgments after which participants cannot explicitly name the objects displayed but solely report having had a “feeling of coherence.” Such findings support the existence of a guiding stage, in which an initial intuitive feeling of coherence is strong enough to trigger a judgment, even if its basis cannot yet be explicitly reported.

**Fig. 1:**
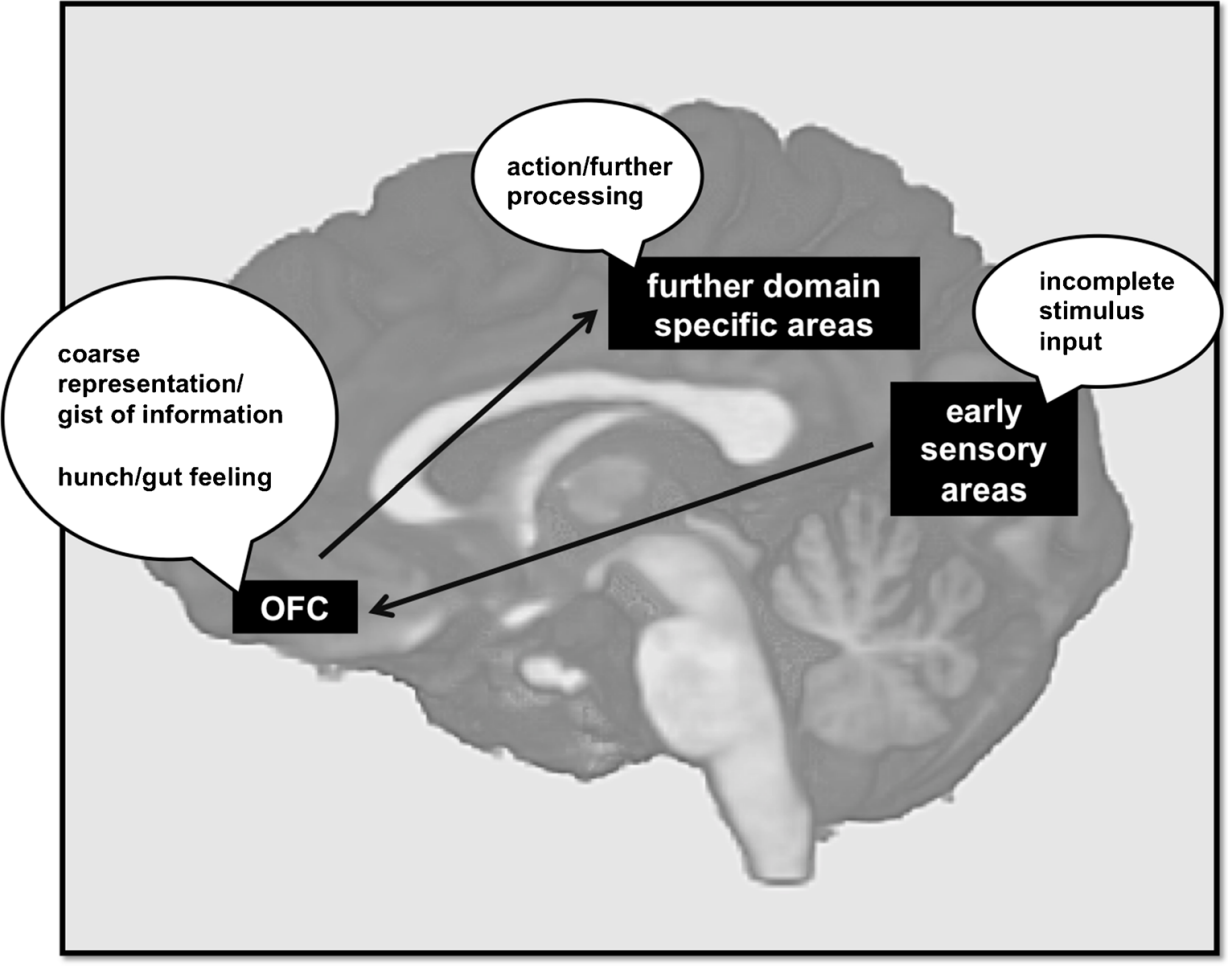
Preliminary neural model of intuitive processing. As proposed by Bar et al. (2006), the orbitofrontal cortex (OFC) functions as an integrator of stimulus input and processes this input toward a coarse representation or gist. We suggest that this coarse representation is, on a subjective level, reflected by an initial hunch or gut feeling that biases judgment and further stimulus analysis. Note that this does not exclude a parallel route directly linking early sensory areas with such responsible for a more detailed analysis (e.g., Plailly, Howard, Gitelman, & Gottfried, 2008). This route however will not be further addressed in the present article

Different neural regions and networks have been suggested to be related to intuitive processing. Satpute and Lieberman (2009), for example, reviewed such literature and, on its basis, proposed a broad neurocognitive model. Regarding research on intuitive judgments related to an initial perception of coherence, however, the orbitofrontal cortex (OFC) sticks out as a crucial structure. In a study combining magnetoencephalography (MEG) and functional magnetic resonance imaging (fMRI), Bar et al. (2006) found the OFC to be involved in the early stages of object recognition. Presenting their participants with hard-to-recognize drawings of nameable objects, they found that OFC activation early after stimulus onset is linked to participants' recognition performance. Furthermore, they found a higher OFC activation for stimuli with low spatial frequency (LSF), in contrast to activation for stimuli with high spatial frequency. LSFs represent the global information about the shape, such as general orientation and proportions, and thus this representation can be thought of as reflecting mostly the gist of the information. On the basis of their results, the authors have suggested that the OFC serves as an initial integrator of incomplete stimulus information and facilitates object recognition by encoding an initial coarse representation containing solely the gist or core idea of the percept. In a top-down process, such a coarse representation may then be signaled onward to structures enabling actions, as well as further, more detailed processing. This conceptualization fits in very well with Bowers et al.’s (1990) framework of intuition, and in the present study, we use it as a preliminary neural model for intuitive processing, as displayed in Fig. 1.

The coarse representation hypothesized to evolve in the OFC would then, on a subjective level, be experienced as an initial hunch or gut feeling that can trigger a coherence judgment, even if the basis of coherence cannot yet be explicitly reported. Studies specifically investigating the neural basis of intuitive processing using WGCT-like paradigms underline such an essential role of the OFC. In one fMRI study, Volz and von Cramon (2006) found activation in the left OFC for the contrast between incomplete stimuli rated as coherent and those rated as incoherent. The authors showed that this activation increase is a function of the probability of a preliminary perception of coherence. Furthermore, the OFC’s role as a detector and predictor of potential content has been suggested to be domain independent, since OFC activation has also been observed for coherence judgments in a WGCT-like task adapted to the auditory domain (Volz, Rübsamen, & von Cramon, 2008). Further supporting evidence for the OFC’s involvement in the representation and top-down processing of gist or meaning has been reported by Luu and colleagues (2010), who used a WGCT-like task with electroencephalography and found that reentrant OFC activation was linked to the judgment of fragmented line drawings as coherent.

The above-mentioned studies provide strong evidence for assuming that the OFC is in some way involved in intuitive judgments of coherence—that is, when the detection of meaning is difficult to grasp and no other, more unambiguous features can be used to help. However, it remains unclear exactly what the role of the OFC is in such judgments. The present study approached this question by testing the plausibility of interpreting OFC activation for intuitive judgments in the framework of the described two-stage model of intuition. Thus, the core research question was: Is it empirically plausible to interpret OFC activation in a WGCT-like task as reflecting the initial intuitive perception of coherence that precedes later stimulus processing geared toward a more explicit understanding?

For this question to be answered in the affirmative, the following five conditions concerning the OFC activation recorded in the present study must be met. (1) To replicate former results and form the basis of the proposed model, an increased OFC activation must appear when participants perceive coherence—that is, when stimuli are judged as coherent (subsequently referred to as *coherent stimuli*), but not when stimuli are judged as incoherent (subsequently referred to as *incoherent stimuli*). (2) As suggested by the results of Bar and colleagues’ (2006) research, but until now not seen in a coherence judgment task, this differential increase in OFC activation must be temporally observed before a differentiation in object recognition areas occurs. Only if condition 2 holds true can one assume that OFC activation reflects an intuitive perception of coherence that, temporally, comes before explicit object recognition. (3) In accordance with Volz and von Cramon’s (2006) results, a significant OFC activation difference must not appear between the different stimulus types as determined objectively (i.e., fragmented and scrambled stimuli). In the performance-dependent contrast (coherent vs. incoherent), we collapse over objective stimulus categories (fragmented and scrambled), aiming to disentangle neural correlates specifically linked to the subjective feeling of coherence. Condition 3 therefore is necessary to remove the concern that OFC activation found for the perception of coherence may be confounded with physical stimulus characteristics—that is, explicable via differential aspects of the two stimulus types that are unrelated to the subjective coherence judgment. (4) A significant OFC activation difference must not be found between scrambled stimuli in experimental versus control trials, with the latter differing only in the preceding task instructions. The control trials, introduced in the present study next to the experimental trials, were marked by a red fixation cross displayed in advance of the stimulus. The red fixation cross indicated that a stimulus did not display a meaningful object and, thus, that no judgment had to be made. Condition 4 is necessary to ensure that OFC activation is not prompted by a participant’s intention—in other words, a participant’s serious attempt to recognize coherence in the line drawings. Such attempts might be assumed to bias participants to judge stimuli as coherent and therefore, like physical stimulus characteristics, influence the contrast between subjectively coherent and incoherent stimuli. However, for control trials, they are completely absent. (5) A final but crucial point that has also not been considered in previous studies is that the differential in OFC activation between subjectively coherent and incoherent stimuli should not depend solely on conscious object recognition. According to Bowers and colleagues’ (1990) model, it is assumed that conscious object recognition is always preceded by a guiding stage—that is, the stage of intuitive processing. If this is correct, time courses of activation in regions related to intuitive processing should be found to be very similar for stimuli for which an object name is given (subsequently referred to as *explicitly coherent stimuli*) and those for which the object cannot be named (subsequently referred to as *implicitly coherent stimuli*). Certainly, to demonstrate that activation in a region is actually representing intuitive processing stages, it is important to show that this activation cannot only be found when implicitly and explicitly coherent stimuli are taken together, but also when explicitly recognized stimuli are not included in the contrast. Proposing the OFC to be involved particularly in the intuitive aspects of stimulus processing, we therefore expect to find no significantly differential activation in the contrast between implicitly and explicitly coherent stimuli. More important, there must be a significant difference in activation of implicitly coherent versus incoherent stimuli. This condition is necessary to rule out the possibility that the OFC may play a role only in explicit recognition, rather than in the early intuitive perception of coherence. Whether these five conditions in support of the proposed model were met was tested with a modified version of the WGCT. MEG was used to record brain activation, because of its high temporal resolution, which is necessary for determining an early involvement of the OFC in coherence judgments.

## Method

### Participants

Twenty-four healthy students (10 males) from the University of Tübingen (age range, 18–29; age mean, 23.583 ± 2.9915) completed the experiment for a payment of 12 Euros per hour. All participants were right-handed, had normal or corrected-to-normal vision, and spoke German as their native language. None had irremovable metal implants in their bodies. The experimental procedure and data collection followed the ethical guidelines of the Declaration of Helsinki (revised version, 2012) and were approved by the local ethical committee of the University of Tübingen Medical Department.

### Stimuli and experimental paradigm

**Fig. 2:**
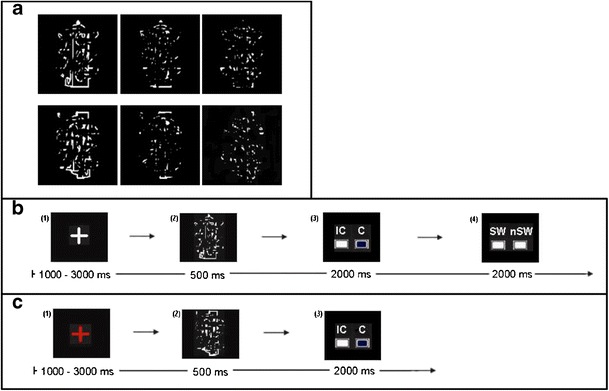
**a** Example of stimuli: fragmented (above) and scrambled (below) line drawings, in three steps of difficulty, from left (lowest level of fragmentation) to right (highest level of fragmentation). Nameable object displayed in the fragmented line drawings is a traffic light; the scrambled line drawings are mixed up versions of the fragmented ones, no traffic light can be recognized anymore. **b** Timeline of an experimental trial. From left to right: (1) fixation cross, (2) stimulus presentation, (3) coherence judgment screen (I = incoherent, C = coherent), (4) solution judgment screen (SW = solution word, nSW = no solution word). Note, that the solution judgment screen appeared only when participants judged a stimulus as coherent. The boxes under the possible answers served as feedback for the participants. When they changed color from white to blue, this indicated that the participant's answer had been recorded. **c** Timeline of a control trial. From left to right: (1) fixation cross, (2) stimulus presentation, (3) buttonpress without decision. The red fixation cross served as an indicator for control trials

Stimuli were taken from the database of Snodgrass figures (Snodgrass & Vanderwart, 1980), which contains a large number of line drawings displaying nameable objects. From those, the stimuli were created as described in Volz and von Cramon (2006). That is, fragmented stimuli were created by removing pixel information via a filter so that the original objects became fairly difficult to recognize. Three levels of fragmentation were defined according to three filters differing in their capacity to mask the object. Scrambled stimuli were created by dividing the fragmented stimuli into eight equally sized parts and randomly mixing those up, so that no meaningful form was left. This procedure ensured that fragmented and scrambled line drawings had exactly the same pixel information and differed only in their higher-order meaning (i.e., their global gestalt). For an example of fragmented and scrambled stimuli and the levels of fragmentation, please see Fig. 2a.

The paradigm used was a modified version of Bowers and colleagues' (1990) WGCT. It was programmed with Presentation® Version 14.8 (Neurobehavioral Systems, Inc.). A timeline for an experimental trial is given in Fig. 2b. On every trial, a line drawing was presented for 500 ms. Subsequently, each participant had 2 s to decide via a buttonpress whether or not the presented line drawing showed a nameable object—that is, whether the stimulus was coherent or incoherent. If participants judged a line drawing as coherent, they had another 2 s to indicate via a buttonpress whether they could actually name the object. Participants were told in the instructions that they would be shown the line drawings they claimed to be able to name (i.e., the explicitly coherent line drawings) again after the experimental session (i.e., outside the MEG). They were also made aware that they would have to write down, in one word, what they thought each of them depicted. Analyzing only the explicitly coherent line drawings, 90.6% (±10.47%) belonged to the objective class of fragmented stimuli, and among these explicitly coherent and fragmented drawings, participants could, outside the MEG, give the correct name or a synonym in 93.08% (±9.09%) of the cases. This link between subjective perception and objective correctness gave us the confidence to assume that participants’ assessment of whether they are able to name a line drawing is based on an actual explicit concept of the object displayed. Note, however, that also the few scrambled line drawings (i.e., those without an objectively correct solution) that were judged as coherent and nameable (median number over participants = 5) were included in the analysis as explicitly coherent stimuli. The same was true for fragmented line drawings that were given a name not reflecting their objectively correct solution. This is because our focus was on the subjective perception of coherence, which does not necessarily need to be equivalent to objective correctness.

Each participant had to make a coherence judgment for 150 fragmented and 75 scrambled line drawings. The levels of fragmentation were distributed equally (50 fragmented stimuli and 25 scrambled stimuli for each level of fragmentation), and all stimuli were fully randomized in sequence. In addition to these 225 experimental trials, there were 60 control trials. The occasional change of task requirements was implemented to control for brain activation involved in basal stimulus processing that is elicited purely from looking at the stimulus without the intention to recognize coherence. Control trials were indicated by a red fixation cross. Subsequently, participants were presented with a control stimulus that always resembled a scrambled line drawing. They received the instruction that line drawings after a red fixation cross would never show a nameable object, so that no attempt at making a judgment had to be made. However, they were asked to pay as much attention to stimuli on control trials as to stimuli on experimental trials. After the scrambled line drawing in a control trial vanished, participants simply had to press one of the two response buttons. A timeline for a control trial is given in Fig. 2c.

### Experimental procedure and recording

The study was conducted at the MEG Center of the University of Tübingen. Participants were seated on a height-adjustable chair inside a magnetically shielded room (Vakuumschmelze, Hanau, Germany). Neuromagnetic recordings were obtained via a 275-sensor, whole-head MEG system (VSM Medtech, Port Coquitlam, Canada). Visual stimuli were projected, using a standard video projector, onto a screen in the recording room that was placed about 60 cm away from the participants.

Participants read the instructions immediately before the beginning of the task, and everyone was asked to repeat the instructions aloud in order for us to determine whether they had been understood. The experimental paradigm took about half an hour. The task began with a training sequence of 12 trials. When participants stated that they understood the task and had no further questions, the experimental blocks started. Participants had to work on 285 trials in five blocks containing 57 trials each. Between blocks, they could take a break, the duration of which they themselves determined. For each individual participant, the 285 presented line drawings were fully randomized in sequence.

The MEG signal was recorded continuously, with a sampling rate of 585.938 Hz. Participants’ head position with respect to sensor positions was measured for every block, and care was taken that head movement remained lower than 5 mm. To ensure that the timing of the measurement was not influenced by the slight delay between the presentation on the computer screen and the projection, each event (i.e., fixation cross, line drawing, answer screen 1, answer screen 2) was encoded via white rectangles in the right corner of the screen. The rectangles were not visible to the participant but were recorded via photo-diodes. Those recordings were later used to determine the exact onset times of events. In addition, participants' responses (coherent/solved = explicitly coherent, coherent/unsolved = implicitly coherent, or incoherent), the sequence of stimulus types (fragmented, scrambled, control), and the according level of difficulty (1, 2, or 3) were recorded by the MEG system via digital triggers.

### MEG analysis

MEG data were analyzed with MATLAB 7.14 (The MathWorks, Natick, MA) and the MATLAB-based software packages Fieldtrip (Oostenveld, Fries, Maris, & Schoffelen, 2011) and Brainstorm (Tadel, Baillet, Mosher, Pantazis, & Leahy, 2011). The Fieldtrip software was used for preprocessing and time-locked averaging over epochs on the sensor level. For preprocessing, the continuous recordings of each block were segmented into epochs of 1.5 s, reaching from 1.000 ms before stimulus onset to 500 ms after stimulus onset, with 200 ms before stimulus onset used for baseline correction. The poststimulus time span encompassed the exact time span during which the line drawings were presented and, therefore, excluded possible confounding of the neuromagnetic responses with the subsequent motor response of buttonpressing. For each block, sensors containing magnetic artifacts (±1 pT) were removed and interpolated; that is, they were replaced by the average of their neighboring sensors. No more than six sensors had to be interpolated in any block. Subsequently, epochs were rejected if they contained magnetic artifacts (±1 pT). No more than 10% of epochs in any of the conditions had to be rejected for any participant. Each participant’s epochs were averaged time-locked to stimulus onset, separately for the conditions of interest (cf. the Results section). The same number of epochs was used for the average of conditions that were planned to be tested against each other (i.e., [1] participants' responses and [2] stimulus types), to make sure that those conditions did not systematically differ in noise level.

On the basis of the time-locked averages, source activation was estimated using the depth-weighted minimum L2 norm estimator of cortical current density (for a detailed description, see Hämäläinen & Ilmoniemi, 1994) as implemented in the Brainstorm software. The source estimation was normalized using the noise covariance matrix (as described by Dale et al., 2000) calculated separately for each participant, over all conditions, from the 1-s prestimulus time span. The underlying forward model was computed using the method of overlapping spheres as described by Huang, Mosher, and Leahy (1999). Given that no individual anatomies from magnetic resonance imaging (MRI) data were available, the forward model was based on the MNI/Colin27 template that is used in the Brainstorm software. The template was warped to fit a set of each participant’s digitized individual head points. For technical details, see Leahy, Mosher, Spencer, Huang, and Lewine (1998).

Differences in source activation between (1) participants' responses and (2) stimulus types were tested via repeated measurement *t*-tests under a significance level of .01. Cluster size and length of time spans served to correct for multiple comparisons. That is, in a first step, differential activation in each vertex on source level averaged over the entire stimulus time span (0–500 ms) was tested for the different stimulus contrasts. To correct for multiple comparisons, only clusters with a *p*-value less than .01 for more than 20 adjacent vertices are reported as significant. In a second step, the mean activation over all vertices of a cluster was used to calculate running *t*-tests at each point in time (each sampling point). Again to correct for multiple comparisons, only time spans with a *p*-value less than .01 for more than 24 consecutive sampling points—that is, time spans of more than 40 ms—are reported as significant.

## Results

### Behavioral results

The behavioral analysis served to make sure that participants were able to—above chance—tell the difference between fragmented and scrambled stimuli and to replicate the finding that the latter was true, even without them being able to explicitly name the objects judged as coherent. In total, participants rated 52.66% (±12.00 percentage points [pp]) of all line drawings (i.e., fragmented as well as scrambled ones) as coherent and claimed to be able to name 27.12% (±10.34 pp) of them. An overview of participants’ mean responses (coherent/incoherent) for the different stimulus types and steps of difficulty is given in Table 1. Applying a signal detection framework, fragmented stimuli rated as coherent are considered hits, given that those stimuli still contain a nameable object to be detected. In contrast, scrambled stimuli rated as coherent are considered false alarms. See Table 2 for an overview over all stimulus types and response alternatives, as well as their interpretation in the signal detection framework. Among fragmented line drawings, the mean percentage of hits was 62.04% (±11.662 pp). The mean percentage of false alarms among scrambled line drawings was 34.01% (±14.419 pp). The parameters *d*′ and *C* were calculated according to signal detection theory (see Abdi, 2007). The parameter *d*′, defined as the difference between the *z*-standardized hit and false alarm rates, was used as a measurement for task difficulty (ranging from 0, which represents chance performance, to infinity, with values higher than 4 representing nearly perfect performance). The parameter *C*, defined as the mean of the *z*-standardized hit and false alarm rates, served to represent response bias (ranging from −1 to 1, with 0 representing unbiased response behavior). The mean *d′* calculated for the present task was 0.775 (±0.259, *p* against zero < .001), which reflects a fairly difficult task, with the difference between signal and noise distribution being slightly lower than one standard deviation of the *z*-statistic (i.e., 1). The mean criterion *C* was 0.064 (±0.350, *p* against zero = .390), which reflects an unbiased response strategy.

**Table 1:** Mean percentage of responses (±*SD*) for the different stimulus categories and steps of difficulty (step 1: most pixel information present, easiest; step 3: least pixel information present, most difficult)

Stimulus Type	Difficulty	Response	
		Coherent (Explicit or Implicit)	Incoherent
Fragmented	step1	81.38 (±10.261)	17.83 (±10.343)
	step2	55.41 (±14.871)	43.95 (±14.466)
	step3	48.99 (±14.572)	50.29 (±14.064)
Scrambled	step1	51.24 (±22.261)	51.27 (±18.976)
	step2	14.44 (±6.405)	70.73 (±12.706)
	step3	13.41 (±8.372)	73.45 (±17.509)

**Table 2:** Overview over all possible combinations of stimulus types and responses, as well as their interpretation in a signal detection framework

		Response		
		Explicitly Coherent	Implicitly Coherent	Incoherent
Stimulus Type	Fragmented	hit (object name provided)	hit (no object name provided)	miss
	Scrambled	false alarm (object name provided)	false alarm (no object name provided)	correct rejection

To check whether participants were able to discriminate between fragmented and scrambled stimuli without explicitly knowing the basis of their discrimination, the difference between hits and false alarms was calculated once again, but after removing explicitly coherent trials. The resulting value has been termed the *intuition index* (Bolte, Goschke, & Kuhl, 2003). In the present study, the percentage of hits neglecting explicitly coherent stimuli was 40.73% (±16.370 pp), and the corresponding percentage of false alarms was 26.84% (±15.388 pp). The intuition index was therefore 13.89% (±7.51 pp), which is significantly over the chance level of zero, *t*(23) = 9.054, *p* < .001.

Mean reaction times of the coherence judgments for the different stimulus types and responses are given in Table 3. A 2 × 3 ANOVA was conducted, with factor one being stimulus type (i.e., fragmented or scrambled line drawings) and factor two being response (i.e., explicitly coherent [judged as coherent and able to name], implicitly coherent [judged as coherent and not able to name], or incoherent [judged as incoherent] stimuli. A significant main effect was revealed of stimulus type, *F*(1, 20) = 11.081, *p* = .003, with fragmented stimuli being decided upon more quickly than scrambled ones. Also, the main effect of response reached significance, *F*(2, 40) = 15.256, *p* < .001, with post hoc tests showing that explicitly coherent stimuli were decided upon more quickly than incoherent stimuli, *F*(1, 20) = 14.165, *p* = .001, as well as more quickly than implicitly coherent stimuli, *F*(1, 20) = 39.168, *p* < .001. A significant interaction effect between the two main factors, *F*(2, 40) = 10.231, *p* < .001, reflected the tendency that in a signal detection framework, correct answers (hits and correct rejections) were given more quickly than incorrect ones (misses and false alarms).

**Table 3:** Mean reaction times (milliseconds after response time onset, ±*SD*) for the different stimulus categories and response types

Stimulus Type	Response		
	Explicitly Coherent	Implicitly Coherent	Incoherent
Fragmented	456.83 (±109.951)	661.53 (±171.082)	660.971 (±169.828)
Scrambled	562.13 (±179.710)	731.26 (±236.822)	616.701 (±165.179)

Altogether, the response pattern and the reaction times indicate that participants processed fragmented stimuli in a different way than they processed scrambled ones and were able to discriminate between them even if they could not explicitly report what this discrimination was based on.

## MEG results

The MEG analysis focused on the five conditions set up to test the plausibility of the proposed neural model of intuitive processing—that is, the notion of the OFC functioning as an early integrator of incomplete stimulus information.

**Fig. 3:**
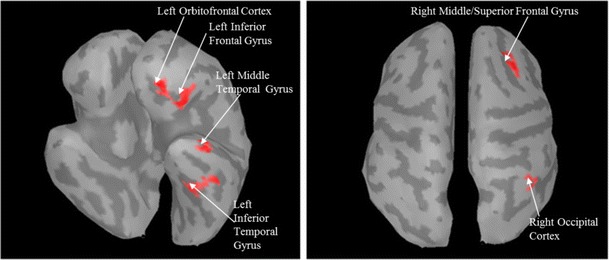
Activated clusters in the performance-dependent contrast. All clusters larger than 20 vertices with significantly (*p* < .01) higher activation (for coherent stimuli, as opposed to incoherent stimuli) are shown

Condition 1 was the basic assumption of stimuli judged as coherent eliciting a higher response in the OFC than stimuli judged as incoherent. Therefore, a performance-dependent contrast (coherent, collapsing explicitly and implicitly coherent vs. incoherent) of MEG activation projected on the source level was calculated for all brain vertices. Six clusters of more than 20 adjacent vertices differing significantly (*p* < .01) between coherent and incoherent stimuli were found, as displayed in Fig. 3. In all of the six clusters, the mean of absolute activation over the entire stimulus time span (500 ms) was higher for coherent stimuli. Clusters were located in (1) the left orbitofrontal cortex, (2) the left inferior frontal gyrus, (3) the left inferior temporal/fusiform gyrus, (4) the left middle temporal gyrus, (5) the right middle/superior frontal gyrus, and (6) the right occipital cortex.

**Fig. 4:**
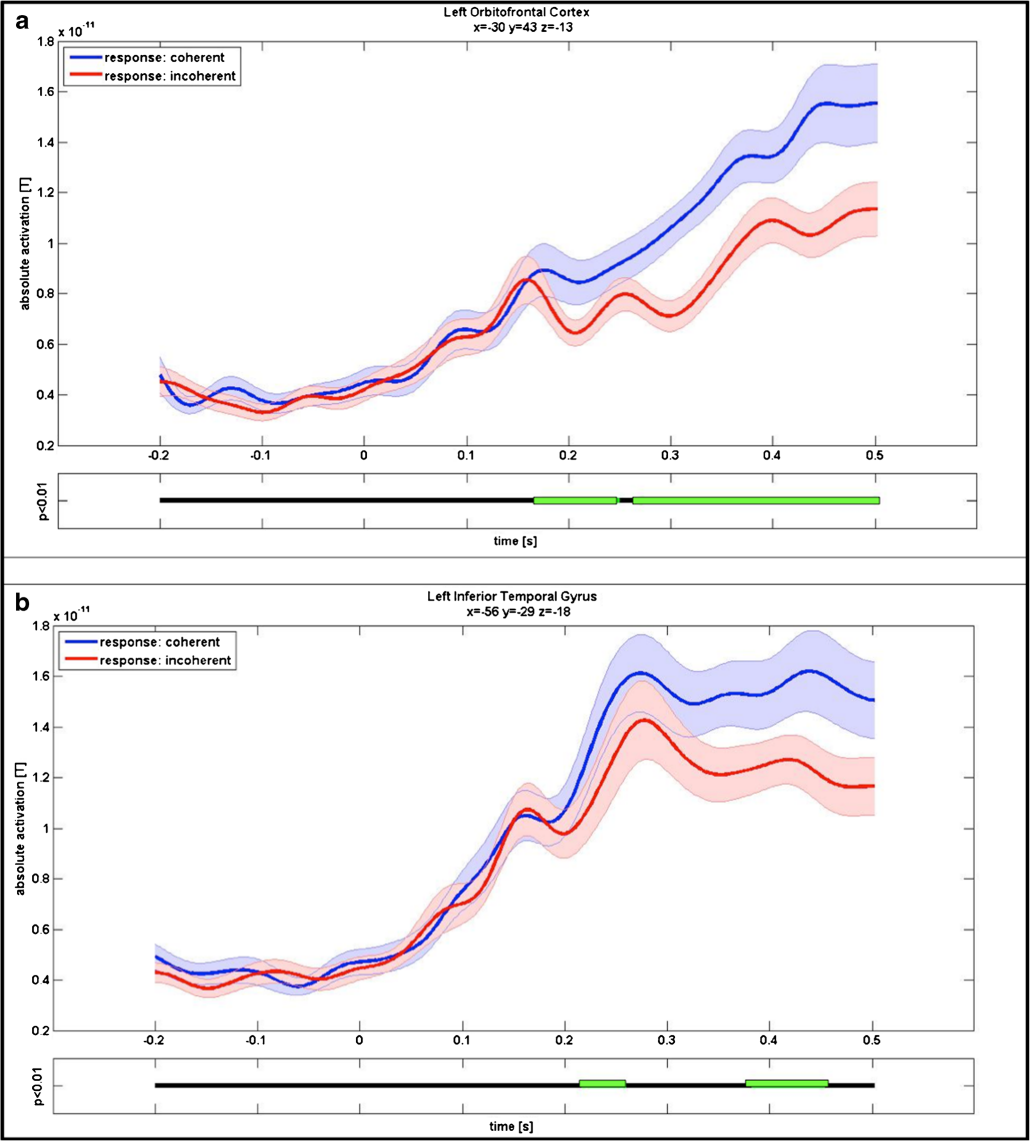
**a** Time courses for the performance-dependent contrast (coherent and incoherent stimuli) in the orbitofrontal cortex. Coordinates *x*, *y*, and *z* indicate the MNI coordinates at the maximal point of differential activation of each cluster. Plotted is the mean of absolute activation over the entire cluster from −200 to 500 ms (with zero being stimulus onset). The blue line signifies coherent trials, the red line incoherent trials; both are surrounded by their standard errors at each point. Time spans significant in repeated measurement *t*-tests (with *p* < .01 for more than 40 ms) are marked in green in the horizontal lines plotted underneath the curves. **b** Time course for the performance-dependent contrast in the inferior temporal gyrus; the figure can be read similarly as in panel a. From panels a and b, it can be seen that the orbitofrontal gyrus differentiation between coherent and incoherent stimuli starts earlier than it does in the inferior temporal gyrus and continues till the end of stimulus presentation

To get an insight into the temporal characteristics of activation and to test condition 2—that is, the OFC activated earlier than object recognition areas—the time courses over stimulus presentation were extracted for all activated clusters. The MNI coordinates, size, and significant time spans (*p* < .01 at every sampling point for more than 40 ms) can be seen in Table 4. Most notably, the time course of the left OFC showed an early significant differentiation starting at 174 ms after stimulus onset and lasting for 70 ms. A second differentiation from 271 ms after stimulus onset till the end of stimulus presentation was found as well. The differentiation at the left inferior temporal/fusiform gyrus was also significant for two time spans, with the first one starting 43 ms later than in the OFC, and the second one starting more than 100 ms later. The time courses for the OFC and the inferior temporal gyrus are shown in Fig. 4a, b. The differentiation in the left inferior frontal gyrus began at 183 ms—that is, later than it did in the OFC, but earlier than it did in the inferior temporal gyrus. The left middle temporal gyrus began differing only at a late time span of 343 ms after stimulus onset and until the end of stimulus presentation. The results presented so far are therefore in accordance with conditions 1 and 2—that is, (1) a substantial involvement of the OFC in the perception of coherence and (2) an early involvement with the OFC being considered to integrate initial stimulus information and signal them onward to further processing areas.

**Table 4:** List of clusters in the performance-dependent contrast: anatomical specifications, MNI coordinates, number of vertices, and significant time spans (milliseconds after stimulus onset) displayed for clusters with higher activation for coherent stimuli than for incoherent stimuli over the mean of stimulus presentation (0–500 ms)

Region	MNI Coordinates			Vertices (Number)	Time Spans (ms)
	x	y	z		
Left orbitofrontal cortex	−30	43	−13	75	174–249
					270–500
Left inferior frontal gyrus	−46	16	−12	64	183–232
					307–500
Left inferior temporal gyrus	−56	−29	−18	88	217–260
					382–454
Left middle temporal gyrus	−64	−3	−17	59	343–500
Right middle/superior frontal gyrus	29	13	60	74	125–183
					304–357
Right lateral occipital cortex	48	−73	36	56	193–302
					384–500

*Note*. All clusters significant over the mean of 0–500 ms with *p* < .01 for more than 20 adjacent vertices are listed. All time spans significant with *p* < .01 for each sampling point in more than 40 ms, as well as the mean of the given time span, are listed for each cluster.

**Fig. 5:**
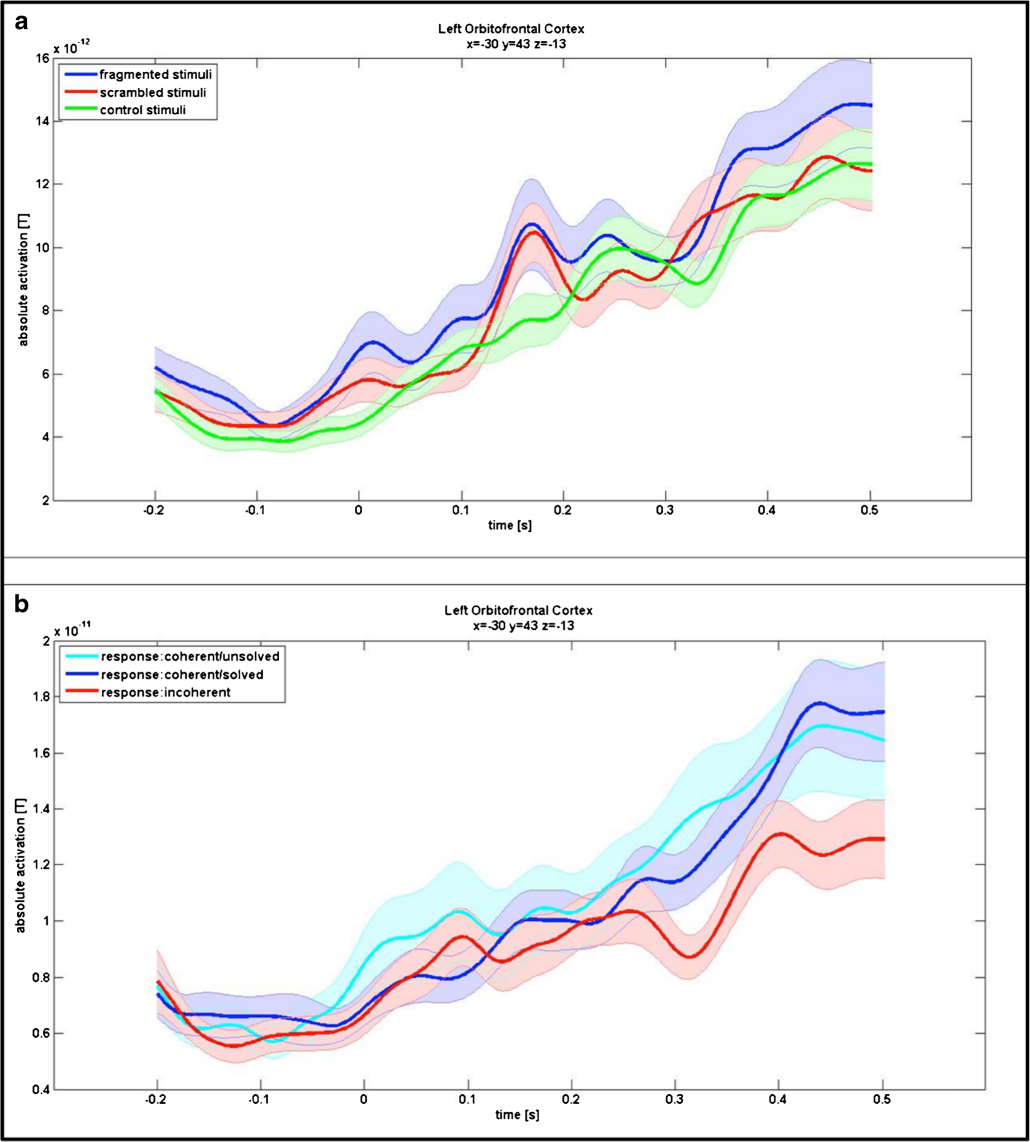
**a** Time courses for the stimulus-dependent contrast (fragmented, scrambled, and control stimuli) in the orbitofrontal cortex. The cluster is taken from the performance-dependent contrast; the plot can be read similarly as in Fig. 4. The blue line signifies trials with fragmented stimuli, the red line trials with scrambled stimuli, and the green line control trials. It can be seen that all three time courses strongly resemble each other and standard errors are highly overlapping. Repeated measurement *t*-tests (scrambled vs. fragmented and fragmented vs. control) did not reveal any differentiation significant for more than 40 ms. **b** Time courses for the performance-dependent contrast, separating coherent trials into implicitly coherent and explicitly coherent ones. The cluster is taken from the contrast, in which unsolved and solved trials were taken together. The cyan line signifies implicitly coherent stimuli, the blue line explicitly coherent stimuli, and the red line incoherent stimuli. It can be seen that the time courses of implicitly and explicitly coherent stimuli are very similar and clearly differentiate from the time course of incoherent stimuli. The latter difference was significant for implicitly coherent stimuli between 269 and 406 ms, as well as 408 and 500 ms, after stimulus onset and for explicitly coherent stimuli between 297 and 426 ms

The purpose of testing conditions 3 and 4—that is, no significant OFC activation differences between fragmented versus scrambled, as well as scrambled versus control, stimuli—was to ensure that OFC activation is specific to the subjective coherence judgments and does not depend on stimulus characteristics or task requirements that might be confounded with the subjective judgment. Therefore, two stimulus-dependent contrasts (fragmented vs. scrambled stimuli and scrambled vs. control stimuli) were investigated. In the stimulus-dependent contrasts. fragmented and scrambled stimuli were collapsed over responses (explicitly coherent, implicitly coherent, or incoherent), so that only the difference between objective stimulus characteristics (3) and task requirements (4) could be investigated and separated from correlates of the subjective feeling of coherence as tested in (1), (2), and (5). No activated cluster could be found anywhere in the orbitofrontal cortex for either of the stimulus-dependent contrasts. Additionally, in extracting time courses for the left OFC cluster as taken from the performance-dependent contrast, there was no significantly different time span for the stimulus-dependent contrasts. The time courses are displayed in Fig. 5a. Conditions 3 and 4 are therewith fulfilled as well, ruling out a stimulus-dependent explanation of OFC activation.

The last condition (5)—that is, increased OFC activation for solely implicitly coherent stimuli—was tested to ensure that the OFC activation found in the contrast between coherent stimuli (collapsed over implicitly and explicitly coherent) and incoherent stimuli does not solely represent explicit object recognition. First of all, contrasting implicitly and explicitly coherent stimuli for the left OFC cluster (taken from the overall performance-dependent contrast of all coherent vs. incoherent stimuli), no significantly different time span could be found. Furthermore, investigating implicitly and explicitly coherent stimuli separately for this cluster, differential activation, as compared with incoherent stimuli, was still present. Between 269 and 406 ms after stimulus onset, as well as 408 and 500 ms after stimulus onset, activation was significantly higher for implicitly coherent stimuli than it was for incoherent stimuli. A difference was also observed for explicitly coherent stimuli in contrast to incoherent ones, although only between 297 and 416 ms after stimulus onset. The time courses of OFC activation in implicitly coherent and explicitly coherent, as contrasted to incoherent, stimuli can be looked up in Fig. 5b. These results fulfill condition 5, ruling out the possibility that OFC activation is explainable via explicit object recognition.

## Discussion

### Study summary and purpose

The present study endeavored to clarify the particular involvement of the OFC in intuitive judgmental processes. Participants were presented with line drawings of either fragmented but still nameable objects or their scrambled counterparts and had to decide for each stimulus whether they believed it was nameable and, if so, whether they could actually name it. Behavioral results in several previous studies of this kind (e.g., Bowers et al., 1990; Luu et al., 2010; Volz & von Cramon, 2006), as well as those in the present study, revealed that participants were able to discriminate above chance between fragmented and scrambled stimuli. Most important, this result also held true when participants stated that they were not able to name the object, which supports the assertion that the present task involves intuitive coherence judgments as defined by Bowers et al. Such intuitive coherence judgments are based on a preliminary hunch arrived at without the actual nature of the coherence in question being reportable, at least at the time of judgment.

Participants’ neuromagnetic responses were recorded with MEG, which, because of its high temporal resolution, is ideal for investigating the temporal dynamics crucial to clarifying the role of OFC activation in intuitive judgments. The proposed preliminary neural model of intuitive coherence judgments, as suggested by Bar and colleagues (2006), conceives of the OFC as an integrator of incomplete stimulus input that signals a first coarse representation of the information forward to further processing areas. We suggest, in line with the idea of Bowers and colleagues (1990), that this coarse representation is experienced, on a subjective level, as an early feeling of coherence that can trigger subsequent judgment and action, even before a full evaluation of the stimulus leads to explicit knowledge of the basis of coherence. If this proposed neural model holds true, OFC activation in the coherence judgment task should then be directly linked to an intuitive feeling of coherence. Earlier results have already provided evidence for the OFC’s involvement in visual coherence judgments (Luu et al., 2010; Volz & von Cramon, 2006). In order for the proposed neural model of the OFC to be valid, certain conditions must hold. (1) OFC activation must increase for stimuli that elicit an intuitive feeling of coherence. (2) The differentiation between subjectively coherent and incoherent stimuli must start earlier in the OFC than in object recognition areas. (3) The differential OFC activation may not be explainable via physical stimulus characteristics; that is, there shall be no OFC activation difference between objective stimulus categories. (4) It shall also not be explainable via task requirements; that is, scrambled and control stimuli where no judgment has to be made shall elicit the same level of OFC activation. (5) OFC activation must reflect the intuitive perception of coherence and not explicit object recognition and, therefore, be independent of whether a stimulus could be explicitly named or not. Our study tested whether those five conditions did in fact hold.

Results confirm the five conditions put up to be in accordance with the present data and, therefore, adding to previous findings, speak to the OFC as an early integrator and, thus, a brain region directly involved in intuitive coherence judgments. These results, at the same time, signal a potential path of study toward a better understanding of the OFC’s specific role in intuitive judgments.

### Condition 1: Orbitofrontal cortex activation is related to an intuitive feeling of coherence

In accordance with earlier research on visual coherence judgments (Luu et al., 2010; Volz & von Cramon, 2006), left OFC activation in the present study was significantly higher for stimuli judged as coherent than for those judged as incoherent. This was shown in a contrast calculated over all vertices on source level, revealing a cluster of 75 adjacent vertices in the left OFC (see Fig. 3). The other six clusters found for this contrast were primarily regions known to be related to object recognition processes. The performance-dependent contrast of coherent (explicitly as well as implicitly) versus incoherent stimuli—with contrasted conditions differing solely in participants' judgments—suggests that OFC activation is related to participants' actual perception of coherence.

### Condition 2: Orbitofrontal cortex activation biases subsequent stimulus processing in object recognition areas

The time courses of presentation for coherent stimuli versus incoherent stimuli revealed a differentiation in the left OFC cluster at 174 ms after stimulus onset, which lasted for 74 ms (see Fig. 4a). This is about 50 ms earlier than the beginning of differentiation in the left inferior temporal/fusiform gyrus (see Fig. 4b), a result that shows that the findings from Bar and colleagues (2006) in their object recognition task also apply for coherence judgments in which complete stimulus information has not been given. The fusiform gyrus is a region of the ventral visual pathway that has been strongly associated with object recognition (e.g., Bar et al., 2001; Grill-Spector & Malach, 2004). The finding that differential activation for coherence perception begins earlier in the OFC than in the fusiform gyrus therefore conforms to the idea of the OFC signaling a coarse stimulus representation forward to domain-specific regions, thereby biasing the judgment toward coherence.

In addition to the inferior temporal/fusiform gyrus, two further regions related to object recognition and naming revealed significant clusters of differential activation over the mean of stimulus presentation: Differential activation in the inferior frontal gyrus began at 183 ms—that is, later than in the OFC and earlier than in the fusiform gyrus. The inferior frontal gyrus has been related to retrieval of semantic relationships (e.g., Bookheimer, 2002; Martin & Chao, 2001) and may function as a crucial link between an initial perception of coherence in the OFC and actual object recognition in the fusiform gyrus. The middle temporal gyrus showed a differentiation starting at 343 ms—that is, only during the second significant time phase in the OFC and the inferior frontal gyrus. Its late involvement is not surprising, considering that this region is especially related to object naming (e.g., Chao, Haxby, & Martin, 1999; Whatmough, Chertkow, Murtha, & Hanratty, 2002), which, logically, is a later step in the object identification process. The finding that the increase of OFC activation temporally occurs before activation in all clusters in the ventral visual pathway (see Table 4 for an overview) is in accordance with its involvement in the initial perception of coherence and furthermore suggests that this initial perception is actually sent onward to areas of visual processing that are involved in subsequent object recognition.

Note that the given data do not imply that OFC involvement in the perception of coherence is limited to signaling onward an initial gist representation. In the present study, differential OFC activation, although beginning early after stimulus onset, actually lasted until stimulus offset. This later activity, as well as the alternating and partly overlapping activity of OFC and object recognition areas, might reflect a more continuous role in stimulus updating, in addition to the proposed initial gist processing. Such an updating of stimulus information on an abstract level is in line with several studies on OFC functionality (e.g., Gluth, Rieskamp, & Büchel, 2012; Peters & Büchel, 2010; Rolls & Grabenhorst, 2008). Similarly, Luu et al. (2010) suggested that reentrant processing dynamics of the OFC are present in visual coherence judgments. Further studies, in which more time is given for stimulus presentation and judgment, are needed in order to investigate exactly how the OFC, in the long run, interacts with other regions involved in coherence perception or in the following steps, like explicit object recognition.

### Conditions 3 and 4: Orbitofrontal cortex activation reflects subjective judgments rather than stimulus characteristics or task requirements

A possible objection to the view that the OFC is reflecting subjective coherence judgments may be that OFC activation could simply be due to stimulus characteristics, rather than to subjective perception. Even when the stimulus types in the present study differed only in the arrangement of their fragments, with low-level stimulus information remaining similar, a difference in processing of the different stimulus types is demonstrated by participants’ over-chance discrimination performance between fragmented and scrambled line drawings. Furthermore, the analysis of reaction times suggested different processes when presented with fragmented, as compared with scrambled, line drawings. Not only were participants significantly quicker in judging fragmented rather than scrambled drawings, but also their results demonstrated a significant interaction between stimulus type and response, which reflects the tendency of “correct” responses (hits and correct rejections) being made more quickly than “incorrect” ones (misses and false alarms). Therefore, the contrast between fragmented and scrambled stimuli collapsed over all response types to only compare physical characteristics of the line drawings was crucial in order to rule out the idea that OFC activation is reflecting general processing differences due to stimulus characteristics. The stimulus-dependent contrast between fragmented and scrambled stimuli, however, did not reveal any significant differentiation within the OFC (see Fig. 5a for the time courses). This supports the notion of the OFC actually reflecting subjective coherence, as opposed to physical differences between stimuli.

Another possible concern may be that OFC activation simply indicates (stronger) attempts at making a decision, which increases the likelihood of finally recognizing coherence. To rule out this objection, we compared scrambled and control stimuli. Scrambled and control stimuli share exactly the same stimulus characteristics; however, they differ in task requirement (i.e., making a decision vs. not making a decision). To disentangle activation reflecting the attempt to make a decision (other than the feeling of coherence or incoherence) and compare it with the lack of this attempt, scrambled stimuli were collapsed over all responses. No significant differentiation between scrambled and control stimuli was found in the OFC (see Fig. 5a). We therefore conclude that OFC activation in the present study reflects the subjective perception of coherence, independently of physical stimulus characteristics and task requirements.

### Condition 5: Orbitofrontal cortex activation reflects an intuitive feeling rather than explicit recognition

A further crucial point that might speak against OFC activation as reflecting the intuitive feeling of coherence would be its dependency on an explicit recognition of the stimuli presented. However, no significant difference could be found in OFC activation between implicitly coherent stimuli, where no explicit recognition had taken place yet, and explicitly coherent stimuli, where a name could be given for the object (supposedly) displayed in the line drawing (see Fig. 5b). Also, taking only implicitly coherent stimuli into account. there was still a significant differentiation of activation in the found OFC cluster. That is, the increase of OFC activation in collapsed coherent, in contrast to incoherent, stimuli was not dependent on explicit object recognition but could be found to a similar degree in response to all stimuli in which a subjective feeling of coherence was elicited (i.e., both implicitly and explicitly coherent stimuli). These observations indicate that OFC activation is involved in processes that convey an unspecific feeling about an object without necessarily triggering explicit recognition. Therefore, the OFC may indeed be crucial to early information accumulation toward an initial feeling of coherence that Bowers and colleagues (1990) suggested to take place in the guiding stage of intuitive judgments. This initial account of the stimulus may then, probably in interaction with further regions, be processed toward a more explicit representation.

### The orbitofrontal cortex is a structure privileged for intuitive processing

The present results, suggesting as they do the essential role of the OFC in intuitive processing, fit very well into the overall understanding that we have at present on this brain structure. The OFC has an unusually high number of anatomical, as well as functional, connections to many different brain areas. It receives information from all sensory modalities (for an overview, see Price, 2006b), which may enable it to function as a global integrator. It has been suggested, though, that the OFC processes sensory information in an abstract way, independently of low-level stimulus characteristics (e.g., O'Doherty & Dolan, 2006). Such coarse and abstract representations may, in fact, be exactly those that create a feeling of coherence that cannot be further explained and, in this sense, may actually establish the basis of intuitive processing.

More than that, the OFC has been shown to have strong interconnections with subcortical structures responsible for emotional behavior and memory functions (i.e., the amygdala, the entorhinal cortex, and the hippocampus), as well as visceral and motor control (i.e., the hypothalamus, the brainstem, and the striatum; Price, 2006b). The former may make an integration of experience and current stimulus information possible, an integration that is necessary for extracting the overall gist of a percept. The latter may enable the triggering of quick behavioral outcomes, with rapidity as a main attribute of intuitive decision making. In sum, all of these characteristics make the OFC a brain structure privileged to play a core role in intuitive processing and in creating an abstract percept that, on a subjective level, leads to an initial feeling of coherence and triggers quick action.

## Conclusion and outlook

The present study endeavored to clarify the role of the OFC in the intuitive perception of coherence. Results suggest that (left) OFC activation in coherence judgments is linked to an initial feeling of coherence that guides subsequent decision and action. Alternative interpretations of OFC activation, reflecting differences in physical stimulus characteristics, task requirements, or explicit object recognition, were ruled out. Such results line up with the proposed neural model of OFC activation as providing an initial coarse representation of the information given. On a subjective level, this coarse representation is thought to be expressed via an initial feeling of coherence.

Future research will have to clarify to what extent the proposed model can be generalized over different domains of coherence judgments (e.g., different sensory domains, semantic judgments). Furthermore, it will be interesting to see whether and how the OFC is integrated in a broader network to enable the representation of an initial feeling of coherence and what specific role it plays in this network. Also, its involvement in an individual’s proceeding from an initial feeling to an explicit understanding will have to be investigated. Furthermore, since the OFC is a highly heterogeneous structure that includes many subregions (cf. Price, 2006a), it will be necessary to differentiate between those in terms of their involvement in intuitive coherence perception by using methods with a higher spatial resolution than MEG. On the basis of the present data, we suggest that the OFC is a structure that cannot be ignored in research that focuses on a neural understanding of intuitive processing and, more specifically, that the proposed neural model may prove a useful starting point for future research in this field.
